# Melatonin Improves Cognitive Function and Suppresses Excessive Mitophagy in Rats With Vascular Dementia

**DOI:** 10.1002/cns.71141

**Published:** 2026-09-03

**Authors:** Si‐jia Hou, Xing‐yi Guo, Zhi‐gang Wang, Ting‐yan Chen, Yang Li, Sui‐yi Xu

**Affiliations:** ^1^ Department of Neurology, Headache Center The First Hospital of Shanxi Medical University Taiyuan China; ^2^ Department of Neurology, Headache Center, Shenzhen Baoan People's Hospital The Second Affiliated Hospital of Shenzhen University Shenzhen China

**Keywords:** chronic cerebral hypoperfusion, cognitive dysfunction, melatonin, mitophagy, vascular dementia

## Abstract

**Aims:**

Melatonin has shown neuroprotective potential in various models of cerebrovascular diseases, but its effects on mitophagy in vascular dementia (VaD) induced by chronic cerebral hypoperfusion (CCH) remain unclear.

**Methods:**

Eighteen adult male Sprague–Dawley rats underwent bilateral common carotid artery occlusion (BCCAO) to induce CCH. Animals were randomly assigned to three groups (*n* = 6 per group): sham‐operated controls, BCCAO model animals receiving vehicle injection, and BCCAO animals treated with daily melatonin (10 mg/kg, intraperitoneally for 14 days). Cognitive performance was evaluated using the Morris water maze. Regional cerebral blood flow (rCBF) was measured with high‐resolution laser Doppler imaging. Mitophagy was assessed via Western blot analysis of LC3BII/I, TOMM20, COX IV, caspase‐3, and immunofluorescence colocalization of LC3B with TOMM20 in the hippocampal CA1 subfield.

**Results:**

BCCAO rats exhibited significant cognitive deficits in the Morris water maze, along with persistently reduced rCBF. BCCAO also induced a significant upregulation of hippocampal mitophagy, as indicated by an elevated LC3BII/I, decreased TOMM20 and COX IV levels, and increased colocalization of TOMM20 and LC3B immunofluorescence signals. Caspase‐3 expression was markedly higher in the BCCAO group. Melatonin treatment improved rCBF recovery at day 14, mitigated the spatial learning and memory impairments, decreased the LC3BII/I, increased mitochondrial protein markers (TOMM20, COX IV), reduced the colocalization of TOMM20 and LC3B puncta, and attenuated caspase‐3 levels.

**Conclusion:**

These findings indicate that CCH in the VaD model induces excessive hippocampal mitophagy and cognitive impairment. The cognitive improvement observed with melatonin treatment is closely correlated with restored cerebral perfusion and attenuated mitophagic activity.

## Graphical abstract

CCH in BCCAO‐operated rats induces excessive hippocampal mitophagy and caspase‐3 upregulation. Two‐week daily melatonin treatment promotes CBF recovery, inhibits excessive mitophagy, and reduces caspase‐3 expression levels. These mechanisms may be associated with melatonin‐mediated amelioration of cognitive dysfunction in VaD rats.

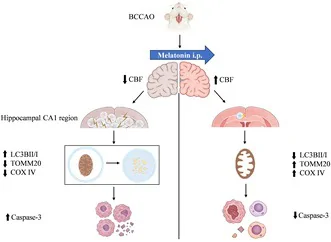

## 1 Introduction

Vascular cognitive impairment (VCI) constitutes a major neurocognitive disorder secondary to cerebrovascular disease and its risk factors, characterized by a broad clinical spectrum of cognitive dysfunction, ranging from subtle subjective cognitive decline to severe vascular dementia (VaD) [1]. With the aging population and rising incidence of cerebrovascular diseases in middle‐aged and older adults, VCI has significantly impaired their quality of life and overall well‐being [2]. VaD represents the advanced, clinically manifest stage of neurocognitive dysfunction within the VCI spectrum [3]. As the second most common cause of dementia, VaD is characterized by complex pathophysiological changes and diverse molecular mechanisms, reflecting its progression from subtle cognitive decline to severe dementia. Chronic cerebral hypoperfusion (CCH) is recognized as a pivotal initiating factor and a consistent pathophysiological hallmark across the spectrum of VCI [2, 4]. The bilateral common carotid artery occlusion (BCCAO) model is widely employed to replicate CCH‐induced cognitive dysfunction, serving as a well‐established experimental paradigm for investigating VCI‐related mechanisms [5].

Mitochondria, as the primary energy‐producing organelles, synthesize ATP via oxidative phosphorylation to sustain cellular homeostasis and activity. Under ischemic–hypoxic conditions, diminished glucose and oxygen availability induce oxidative phosphorylation uncoupling, impair mitochondrial respiratory chain integrity, and compromise mitochondrial membrane potential, thereby exacerbating cellular metabolic dysfunction. This initial metabolic perturbation triggers a cascade of secondary disturbances, including heightened oxidative stress, aberrant inflammatory signaling, and calcium ion dysregulation, which collectively converge to activate apoptotic signaling pathways [2]. Mitophagy represents a selective autophagic process dedicated to the clearance of dysfunctional mitochondria [6]. Within the mitochondrial quality control system, three principal signaling pathways orchestrate mitophagy: the PTEN‐induced putative kinase 1 (PINK1)/Parkin pathway, the BCL2 interacting protein 3 like (BNIP3L/NIX) pathway, and the FUN14 domain containing 1 (FUNDC1) pathway [6]. Under conditions of oxidative stress, PINK1 accumulates on the outer mitochondrial membrane (OMM), subsequently recruiting Parkin from the cytosol to the OMM [7]. Activated Parkin catalyzes the ubiquitination of multiple OMM proteins, generating polyubiquitin chains that serve as molecular tags for damaged mitochondria [8, 9]. This ubiquitination event triggers the recruitment of microtubule‐associated protein 1A/1B light chain 3 (LC3), which facilitates the selective engulfment of ubiquitinated mitochondria into autophagosomes for subsequent degradation. The autophagosomes subsequently undergo fusion with lysosomes to form autophagolysosomes, wherein the damaged mitochondria are ultimately degraded through lysosomal enzymatic activity [10]. This process culminates in the selective degradation of both inner and outer mitochondrial membrane proteins, thereby completing the mitophagy pathway [11]. Regarding the BNIP3L/NIX and FUNDC1 pathways, these pathways contain conserved LC3‐interacting regions within their structural domains, enabling direct recognition and binding to LC3 to facilitate mitophagy [12, 13]. However, mitophagy exhibits a dual role in cerebral ischemia, with its functional impact remaining debated [14]. In the context of VaD, the dynamic alterations in mitophagy during prolonged ischemia and hypoxia across disease progression remain incompletely characterized [15]. A prior study demonstrated that hippocampal mitophagy was significantly upregulated in rats 5 weeks post‐BCCAO surgery, correlating with increased apoptosis [16]. While moderate mitophagy promotes neuronal survival, both excessive and deficient mitophagy contribute to cellular injury and subsequent cell death [17]. Modulating mitophagy activity, either through inhibition or enhancement, may help restore mitochondrial homeostasis and metabolic balance, ultimately reducing neuronal loss [11].

Melatonin (N‐acetyl‐5‐methoxytryptamine, Mel) is a pleiotropic hormone that orchestrates circadian rhythm synchronization and regulates diverse physiological processes. Endogenously synthesized by the pineal gland, its secretion is dynamically controlled by the light/dark cycle through neural input from the suprachiasmatic nucleus, resulting in rhythmic release into the systemic circulation [18, 19]. In the context of ischemia–reperfusion injury and neurodegenerative disorders, the therapeutic potential of melatonin in modulating mitochondrial oxidative stress responses and mitophagy has been extensively investigated in preclinical models [20]. While prior investigations in VaD models have demonstrated that melatonin mitigates cognitive impairment through attenuation of oxidative stress, inflammatory signaling, endoplasmic reticulum stress, blood–brain barrier (BBB) dysfunction, and reduced apoptotic cell death [21, 22], the potential regulatory effects of melatonin on mitophagy in VaD pathogenesis remain to be elucidated.

## 2 Methods

### 2.1 Animal Preparation

**FIGURE 1:**
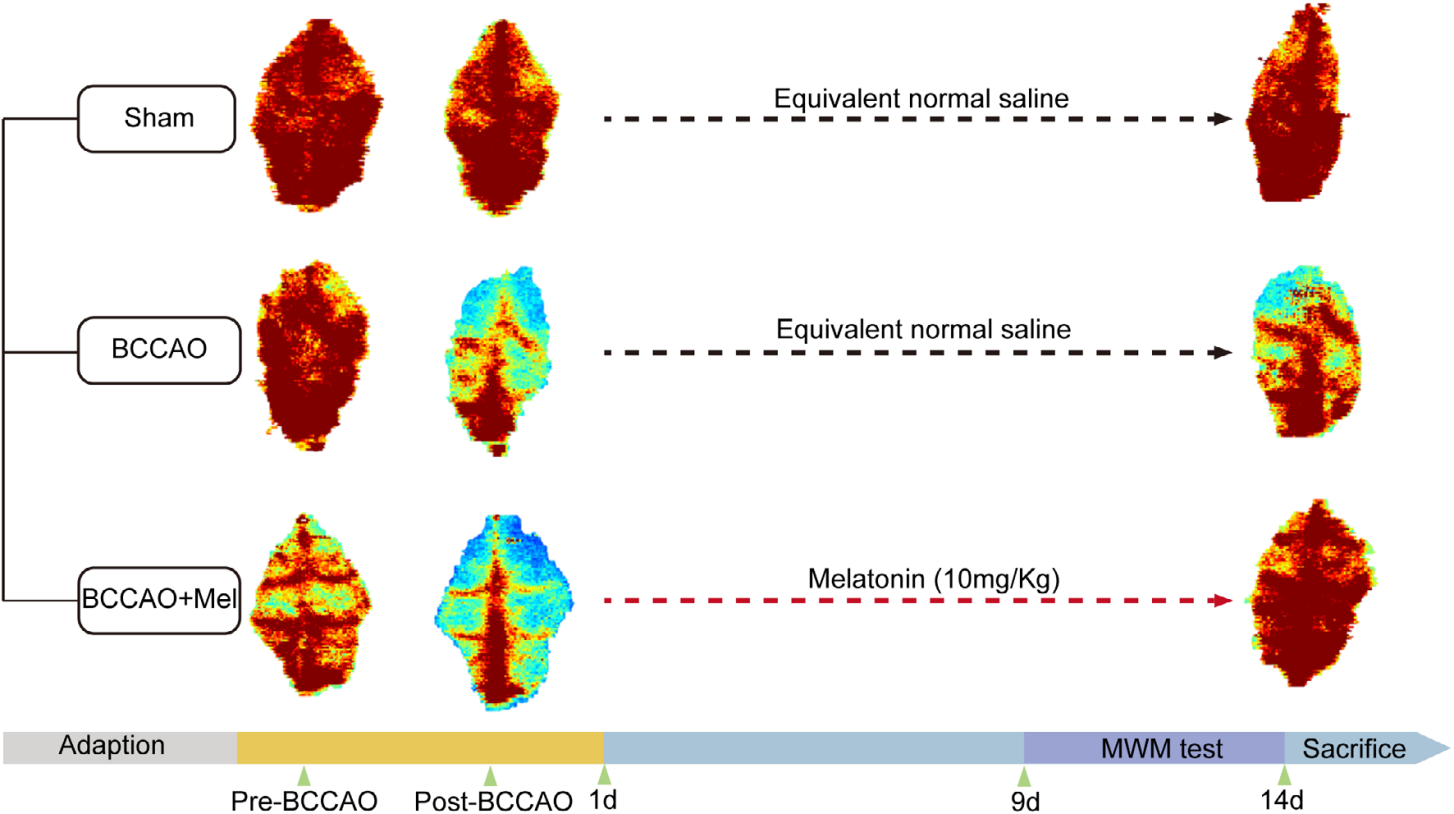
Experimental flow chart and the temporal profile of regional CBF in each group. “Pre‐BCCAO” indicates baseline; 1d, day 1 post‐surgery; 9d, day 9 post‐surgery; 14d, day 14 post‐surgery.

Eighteen male Sprague–Dawley rats (body weight: 230 ± 20 g, age: 6 weeks) were obtained from the Experimental Animal Center of Shanxi Medical University. The housing conditions were meticulously maintained to ensure animal welfare, with a controlled temperature of 22°C ± 2°C, relative humidity of 50% ± 5%, and a standardized 12‐h light/dark cycle. Animals had ad libitum access to standard rodent chow and filtered water throughout the experimental period. Sprague–Dawley rats (*n* = 18) were randomly allocated to three experimental groups (*n* = 6 per group): (1) Sham‐operated controls receiving vehicle injection (Sham group); (2) BCCAO model animals receiving vehicle injection (BCCAO group); and (3) BCCAO model animals receiving daily melatonin administration (BCCAO + Mel group) (Figure 1). A total of twenty‐three male Sprague–Dawley rats were initially enrolled, of which five were excluded due to surgical complications or postoperative death. The reasons were as follows: one rat died of uncontrollable hemorrhage from the common carotid artery due to a surgical error during BCCAO; one sham‐operated rat developed progressive abdominal distension postoperatively and was found to have extensive intestinal dilation upon autopsy; two BCCAO rats and one BCCAO + Mel rat ceased eating and drinking postoperatively and subsequently died. Consequently, the remaining eighteen rats completed the study and were included in the data analysis. Three rats (one per group) that developed unilateral narrowing of the palpebral fissure after surgery, likely suggestive of Horner's syndrome due to inadvertent cervical sympathetic chain injury, were excluded from behavioral testing. This did not compromise their suitability for the molecular assays. Thus, three rats per group were used for Western blotting and the other three for immunofluorescence, as the two techniques require different tissue processing (rapid freezing versus paraformaldehyde perfusion).

### 2.2 BCCAO Surgery and Drug Administration

BCCAO surgery was performed following established surgical protocols [23, 24]. Animals were anesthetized via intraperitoneal administration of sodium pentobarbital (40–50 mg/kg) and placed in a supine position to ensure complete exposure of the cervical region. Following hair removal and aseptic preparation of the surgical site, a midline longitudinal incision measuring approximately 2 cm was created in the cervical region. Under stereomicroscopic visualization, the bilateral common carotid arteries were meticulously dissected free from the vagus nerve, fully exposed, and permanently ligated with 5–0 silk sutures. The wound was subsequently closed with sutures and subjected to antiseptic treatment. In sham‐operated controls, the identical surgical protocol was performed with the exception of bilateral common carotid artery ligation. Core body temperature was carefully regulated throughout the surgical procedure and during the post‐anesthetic recovery period. Animals in the BCCAO + Mel group received daily intraperitoneal injections of melatonin (10 mg/kg dissolved in 0.9% saline; Beyotime Biotechnology, China) [25], commencing on the first postoperative day. In parallel, both the Sham and BCCAO groups were administered equivalent volumes of normal saline via the same route and schedule. All treatments were consistently maintained for 14 consecutive days, with each administration completed prior to 08:00 daily. All daily intraperitoneal injections were conducted by an investigator blinded to group allocation and treatment regimens.

### 2.3 Regional Cerebral Blood Flow (rCBF) Measurement

To validate the success of BCCAO surgery and track dynamic alterations in cerebral hemodynamics, rCBF measurements were obtained using a high‐resolution laser Doppler perfusion monitoring system (Moor LDI2‐HIR, Moor Instruments, UK) [26]. Following anesthesia, rats were immobilized in a stereotaxic apparatus in the prone position. After depilation and aseptic preparation of the scalp, a midline vertical incision approximately 2 cm in length was made and extended bilaterally through blunt dissection. The skull surface was treated with 3% hydrogen peroxide to remove periosteal tissue surrounding the bregma, followed by thorough drying with sterile cotton swabs to eliminate residual moisture. rCBF images of the exposed skull region were acquired through laser Doppler scanning. A circular region of interest (ROI) with a 10 mm diameter centered on the bregma was defined for quantitative analysis. Mean rCBF values within the ROI were documented for five animals per group at three distinct intervals: preoperatively, 1 h post‐surgery, and 14 days post‐surgery (after the Morris water maze test). All rCBF measurements were normalized as percentages relative to the mean values of the Sham group at corresponding time points. The analysis of rCBF was performed by an investigator blinded to the experimental groups.

### 2.4 Morris Water Maze (MWM) Test

The MWM assessment was initiated on postoperative day 9 to evaluate spatial learning and memory across experimental groups, following a previously established protocol [27]. The experimental timeline is schematically presented in Figure 1. The MWM apparatus comprises a circular black pool (120 cm in diameter, 60 cm in depth) integrated with an automated video‐tracking system. All behavioral testing was conducted in a controlled environment maintained at 23°C ± 2°C with minimal ambient disturbance. The circular swimming pool was partitioned into four equal quadrants by an imaginary “+” grid, with a circular platform (10 cm diameter) positioned in the center of the southwest (SW) quadrant. The platform was submerged approximately 2 cm below the water surface, achieved by adding edible black dye to the water. Distinct spatial cues were positioned on the inner walls of the pool corresponding to each quadrant. Individual rats were carefully introduced into the water by the experimenter assigned to their daily husbandry and pharmacological interventions. The duration required for each animal to locate the submerged platform was quantified as escape latency. Rodents were permitted unrestricted exploration for 120 s; unsuccessful platform localization within this period resulted in trial termination, whereupon animals were manually guided to remain on the platform for 10 s. During the 5‐day acquisition phase, all animals completed four daily trials initiated from distinct entry points. On day 6, the spatial probe test was conducted with the platform removed. Each rodent was released from the northeast (NE) quadrant with unrestricted access to investigate the former platform location. The following behavioral metrics were systematically quantified throughout testing: escape latency, platform crossings, quadrant occupancy duration, swimming velocity, and navigation trajectories. The MWM test was conducted and recorded by two investigators who were blinded to the group allocation.

### 2.5 Western Blot

On the day of the final rCBF measurement, the rats were sacrificed, and hippocampal tissue was collected from each rat brain, immediately frozen in liquid nitrogen, and stored at −80°C. Cold RIPA buffer (Boster, China) supplemented with PMSF (Boster, China) was used to lyse the tissue on ice. After centrifugation, the supernatant was collected, and protein concentration was determined using a BCA Kit (Boster, China). Proteins (30 μg) were separated by 12% SDS‐PAGE (sodium dodecyl sulfate‐polyacrylamide gel electrophoresis) and subsequently transferred onto 0.22 μm pore‐size polyvinylidene fluoride (PVDF) membranes using a semi‐dry transfer system (Bio‐Rad, USA). The PVDF membranes were blocked with 5% non‐fat milk (Boster, China) at room temperature for approximately 2 h. Following removal of residual milk, the membranes were incubated overnight at 4°C with appropriately diluted primary antibodies, followed by incubation with diluted secondary antibodies (1:5000, SE134, Solarbio, China) at room temperature for 2 h. After immersion in ECL solution (Boster, China) for 30 s, the protein bands on membranes were visualized using an imaging system (Bio‐Rad, USA) and quantified using ImageJ software (National Institutes of Health, USA). The following primary antibodies were used in our experiment: LC3B (1:2000, ab192890, Abcam, UK), translocase of outer mitochondrial membrane 20 (TOMM20, 1:1000, BM4366, Boster Biological Technology, China), cytochrome c oxidase IV (COX IV, 1:5000, #4850, Cell Signaling, USA), caspase‐3 (1:1000, A2156, Abclonal, China), and β‐Actin (1:5000, AC026, Abclonal, China). The analysis of Western blot bands was performed by an investigator blinded to the experimental groups.

### 2.6 Immunofluorescence Staining

After the final rCBF measurement, anesthetized rats underwent intracardiac perfusion with 0.9% saline, followed by 4% paraformaldehyde in phosphate buffer. The brains were then removed and post‐fixed by immersion in 4% paraformaldehyde at 4°C overnight. Subsequently, the tissues were cryoprotected by sequential immersion in 15% and 30% sucrose solutions. Finally, the brains were carefully stored at −80°C for long‐term preservation. The brain tissue, embedded in optimal cutting temperature compound, was sectioned coronally into 20‐μm‐thick slices using a freezing microtome (Leica, Germany). To prevent detachment during subsequent experimental procedures, the slices mounted on adhesive‐coated slides were baked at 65°C for 1 h. Following antigen retrieval with EDTA‐Tris buffer (pH 9.0) (ZSGB‐BIO, China), tissue sections were rinsed three times with phosphate‐buffered saline (PBS). They were subsequently permeabilized with PBS containing 0.2% Triton X‐100 (Solarbio, China) and blocked with 5% bovine serum albumin. Primary antibody incubation was performed overnight at 4°C in a humidified chamber. On the following day, tissue sections were washed three times (10 min per wash) with PBS containing 0.1% Tween‐20. Subsequently, they were incubated with fluorescence‐conjugated secondary antibodies at room temperature for 1.5 h. Nuclei were counterstained with 4′,6‐diamidino‐2‐phenylindole (DAPI; Beyotime, China). The following primary antibodies and dilutions were employed for immunostaining: rabbit anti‐LC3B (1:500, Cat. #ab192890, Abcam, UK) and rabbit anti‐TOMM20 (1:500, Cat. #ab283317, Abcam, UK). For immunofluorescence detection, the following fluorescence‐conjugated secondary antibodies were applied at a dilution of 1:300: Alexa Fluor 594 (Cat. #ZF‐0516, ZSGB‐BIO, China) and Alexa Fluor 488 (Cat. #ZF‐0512, ZSGB‐BIO, China). Immunofluorescence was visualized and images were acquired using a fluorescence microscope (OLYMPUS, Japan). For analysis, three random fields of view were captured from the hippocampal CA1 region at 200× magnification. Colocalization of LC3 with the mitochondrial marker TOMM20, indicative of mitophagy, was quantified using the ImageJ software (National Institutes of Health, USA). Quantification of colocalization and fluorescence intensity was conducted by an observer blinded to the experimental design.

### 2.7 Statistical Analysis

Data are presented as the mean ± standard deviation (SD). Statistical analyses were conducted primarily using one‐way analysis of variance (ANOVA) with Tukey's honest significant difference (HSD) post hoc test in GraphPad Prism, version 10.1. The exception was the analysis of group differences in escape latency across the five training days, for which repeated‐measures ANOVA followed by Bonferroni post hoc correction was applied using SPSS, version 26.0. Statistical significance was defined as a two‐tailed *p* value of less than 0.05 (*p* < 0.05).

## 3 Results

### 3.1 Melatonin Promoted rCBF Recovery in BCCAO Rats

**FIGURE 2:**
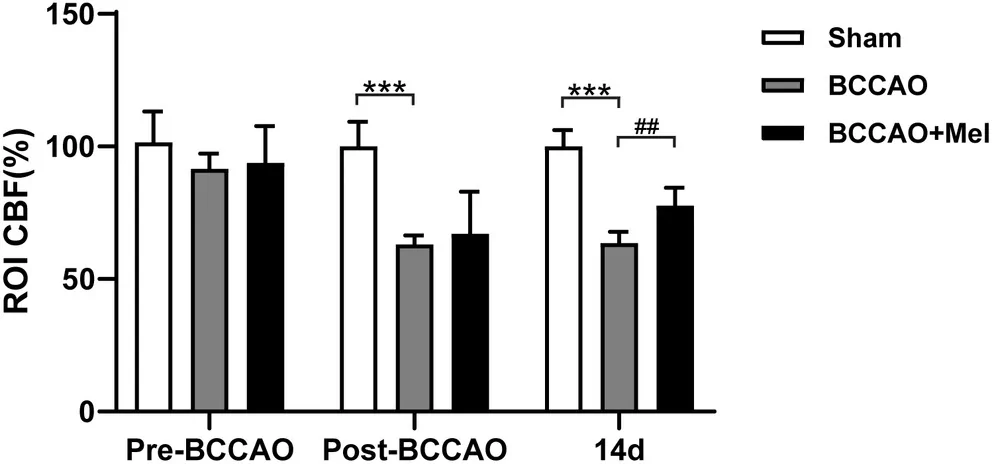
Mel promoted CBF recovery in BCCAO rats, as examined by a high‐resolution laser Doppler (*n* = 5). ***p < 0.001, ^##^ *p* < 0.01. CBF, cerebral blood flow. “Pre‐BCCAO” indicates baseline; 14d, day 14 post‐surgery.

rCBF in the frontal and parietal cortices of rats was quantified using high‐resolution laser Doppler imaging. As illustrated in Figures 1 and 2, the mean rCBF within the ROI was significantly reduced in the BCCAO and the BCCAO plus melatonin (BCCAO+Mel) treatment groups compared to the Sham group (BCCAO: 63% ± 3.4%; BCCAO+Mel: 67% ± 1.6%). Furthermore, no statistically significant difference was observed between the two groups (*p* > 0.05), suggesting that the initial severity of cerebral ischemia was comparable. By day 14 post‐occlusion, the mean rCBF in the ROI had increased to 78% ± 6.8% in the BCCAO+Mel group. This value was significantly higher than that in the BCCAO‐only group (64% ± 4.3%; *p* < 0.01), as shown in Figure 2. These results indicate a potential role of melatonin in promoting the recovery of cerebral perfusion in the chronic phase following BCCAO.

### 3.2 Melatonin Alleviated Spatial Learning and Memory Impairments in BCCAO Rats

**FIGURE 3:**
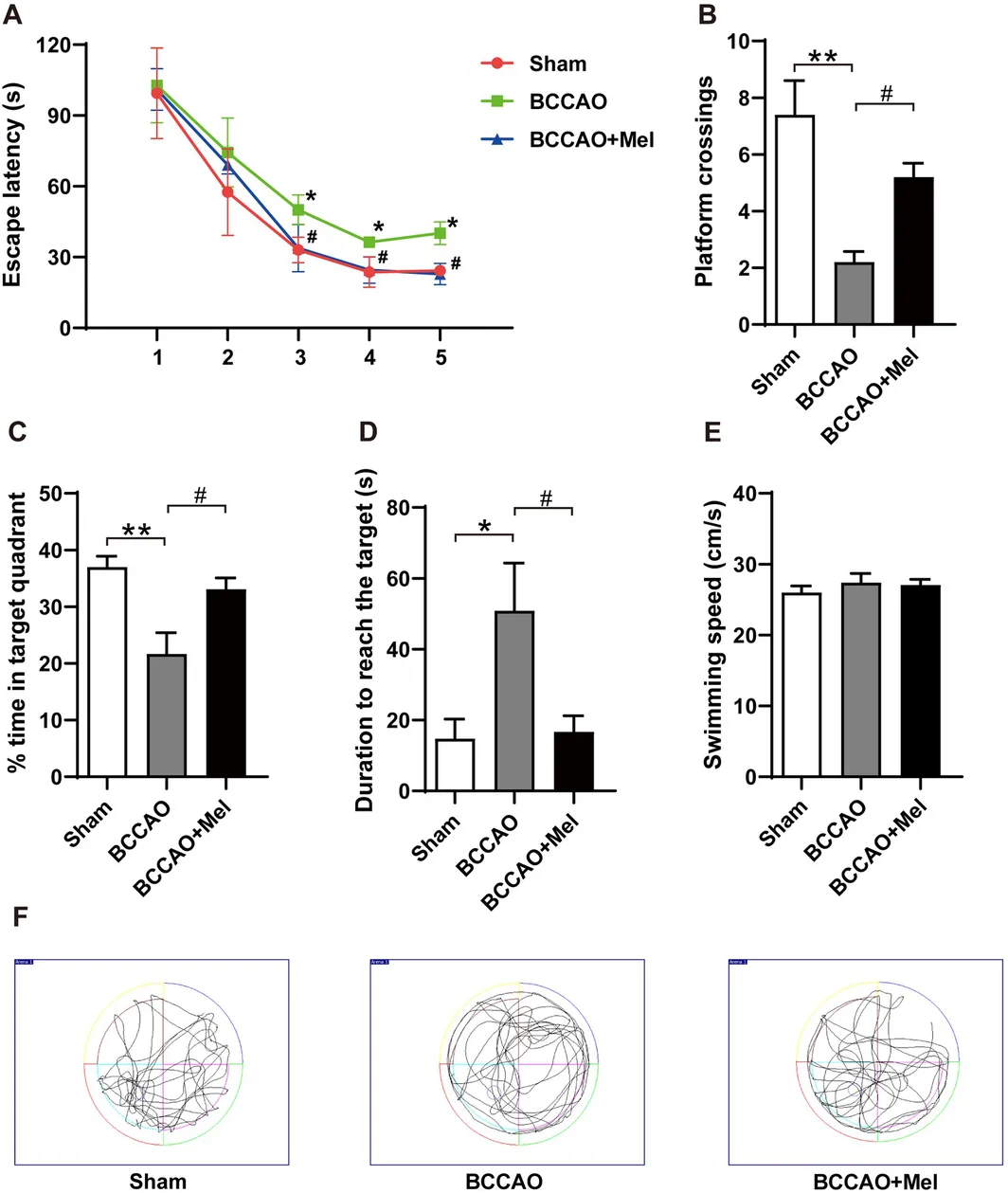
Mel alleviated spatial learning and memory impairments in BCCAO rats, as measured by the MWM test (*n* = 5). (A) Escape latency changes of the three groups in the first 5 training days. (B) Platform crossings on day 6. (C) Percentage of time spent in platform quadrant on day 6. (D) Duration to reach the target location on day 6. (E) Swimming speed on day 6. (F) Representative swimming paths from each group on day 6. *p < 0.05, **p < 0.01, ^#^ *p* < 0.05.

Spatial learning and memory abilities in rats were assessed using the MWM test to compare cognitive performance across experimental groups. As presented in Figure 3A, rats subjected to BCCAO demonstrated significantly prolonged escape latencies and required more time to locate the hidden platform compared to animals in the other two groups. In the escape latency, there was a significant effect over time (*F*[2.599, 31.193] = 161.779, *p* < 0.001, *η*_*p*_^2^ = 0.931) and also a significant effect for treatment (*F*[2, 12] = 7.606, *p* < 0.01, *η*_*p*_^2^ = 0.559). The interaction of treatment and time was not significant (*F*[5.199, 31.193] = 0.976, *p* > 0.05, *η*_*p*_^2^ = 0.14). On training days 3, 4, and 5, both the Sham and BCCAO+Mel groups required significantly less time to locate the hidden platform compared to the BCCAO group, as reflected by their shorter escape latencies (*p* < 0.05). Throughout the acquisition phase of the training, no statistically significant difference in escape latency was detected between the BCCAO+Mel and Sham groups (*p* > 0.05; Figure 3A). During the probe trial conducted on day 6 (Figure 3F), rats in the BCCAO group exhibited significant impairments in spatial memory retention. In contrast, animals receiving melatonin treatment (BCCAO+Mel group) performed comparably to the Sham‐operated controls, with no statistically significant difference detected between these two groups. The memory deficit in the BCCAO group was characterized by multiple indicators: a reduced number of platform‐site crossings (*F*[2, 12] = 11.11, *p* < 0.01; Figure 3B), a lower percentage of time spent searching in the target quadrant (*F*[2, 12] = 8.765, *p* < 0.01; Figure 3C), an increased latency to first entry into the former platform location (*F*[2, 12] = 5.35, *p* < 0.05; Figure 3D), and fewer entries into the target quadrant itself (*F*[2, 12] = 7.505, *p* < 0.01; Figure S1). Melatonin treatment significantly attenuated these cognitive deficits (*p* < 0.05). The mean swimming speeds were comparable across all experimental groups (*F*[2, 12] = 0.462, *p* > 0.05; Figure 3E), indicating that the observed cognitive impairments were not confounded by deficits in locomotor or motivational function. Collectively, these findings suggest that melatonin administration is effective in mitigating the cognitive dysfunction induced by CCH in the BCCAO model.

### 3.3 Melatonin Inhibited Excessive Mitophagy in BCCAO Rats

**FIGURE 4:**
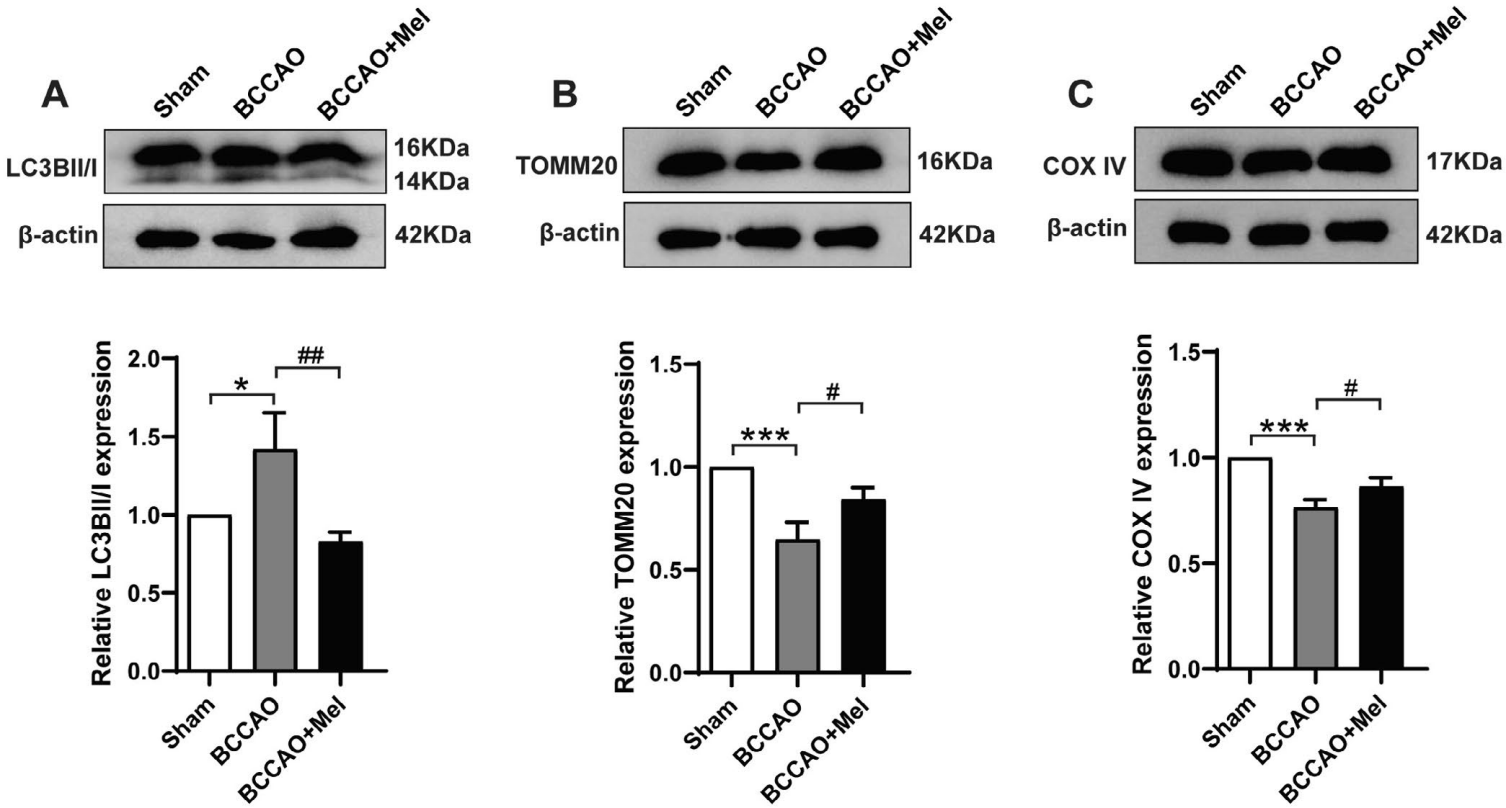
Mel inhibited excessive mitophagy in BCCAO rats, as examined by Western blot (*n* = 3). (A–C) The Western blot band and quantification of the relative expression of LC3B II/I, TOMM20, COX IV. *p < 0.05, ***p < 0.001, ^#^ *p* < 0.05, ^##^ *p* < 0.01.

**FIGURE 5:**
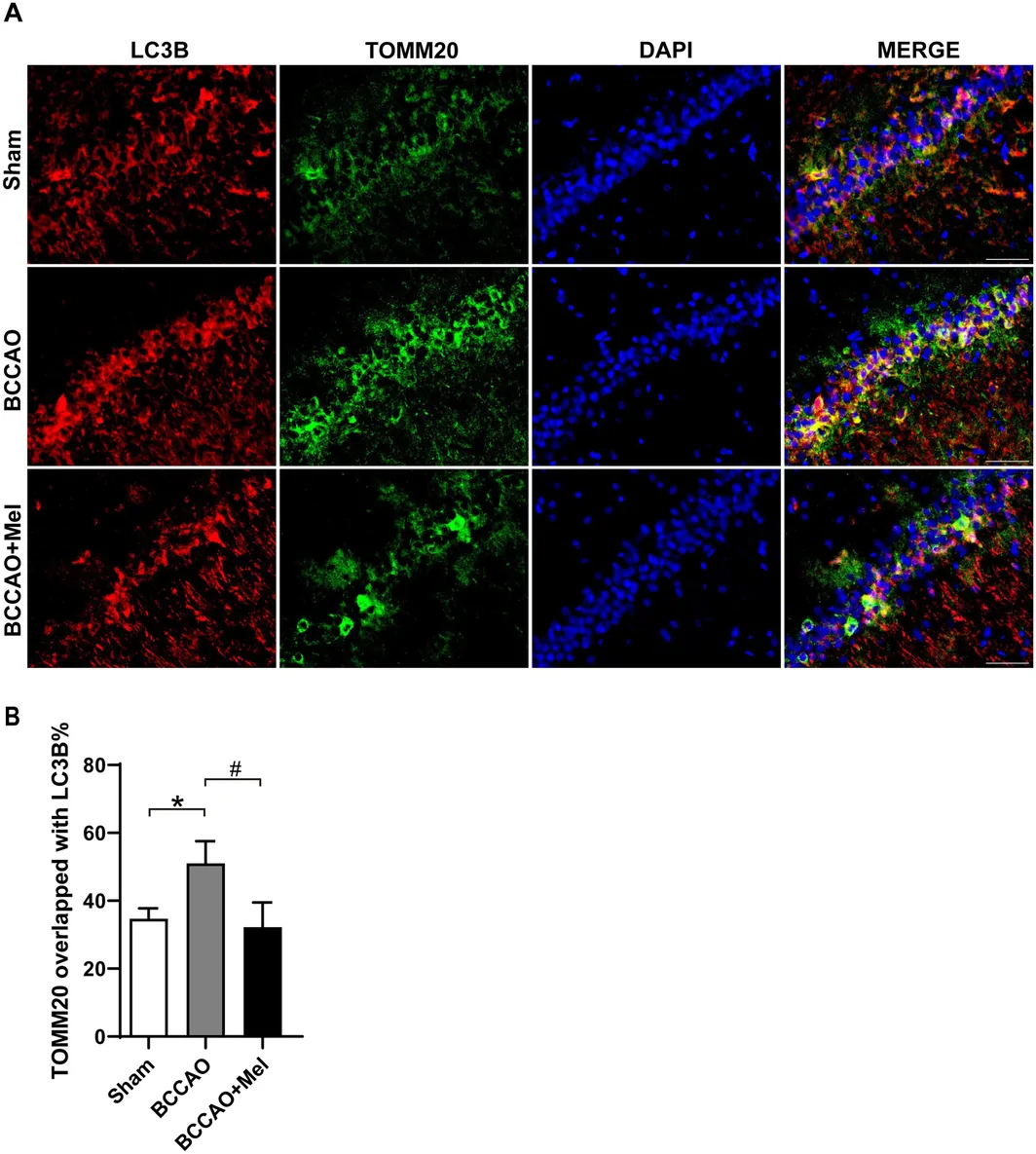
Mel downregulated excessive mitophagy in BCCAO rats, as detected by immunofluorescence (*n* = 3). (A) Representative images of LC3B and TOMM20 colocalization observed in the hippocampal CA1 area. (B) The ratio of TOMM20 overlapping with LC3. *p < 0.05, ^#^ *p* < 0.05. Scale bar = 50 μm, 200×.

To investigate the potential regulatory role of melatonin on mitophagy in the hippocampus following CCH, we employed Western blot analysis to quantify the protein expression levels of key markers in hippocampal tissue: the autophagosome‐associated protein LC3B, the mitochondrial outer membrane translocase TOMM20, and the inner membrane respiratory complex subunit COX IV. Compared to the Sham‐operated controls, rats subjected to BCCAO exhibited a significant increase in the ratio of LC3B‐II to LC3B‐I in the hippocampus (*p* < 0.05; Figure 4A). Conversely, the hippocampal protein levels of the mitochondrial markers TOMM20 (*F*[2, 6] = 27.15, *p* < 0.05; Figure 4B) and COX IV (*F*[2, 6] = 41.21, *p* < 0.001; Figure 4C) were markedly lower in the BCCAO group. Together, these molecular alterations are consistent with an enhancement of mitophagic activity in the hippocampus following CCH. To further assess mitophagy at the cellular level, immunofluorescence co‐staining for the mitochondrial marker TOMM20 and the autophagosomal marker LC3B was performed in the hippocampal CA1 subfield. The presence of overlapping punctate fluorescence signals for TOMM20 and LC3B within individual neurons was interpreted as evidence of mitophagic events [28]. In alignment with the immunoblotting findings, immunofluorescence analysis revealed a greater extent of colocalization between TOMM20 and LC3B puncta in the hippocampal CA1 region of the BCCAO group compared to Sham‐operated controls (*p* < 0.05, Figure 5B). This increased colocalization signal, indicative of elevated mitophagic activity, was attenuated in rats receiving melatonin supplementation (BCCAO+Mel group) (Figure 5A,B). Collectively, these data suggest elevated mitophagy in the CA1 subfield triggered by CCH, and melatonin treatment may effectively counteract this excessive autophagic response.

### 3.4 Melatonin Reduced Hippocampus Caspase‐3 in BCCAO Rats

**FIGURE 6:**
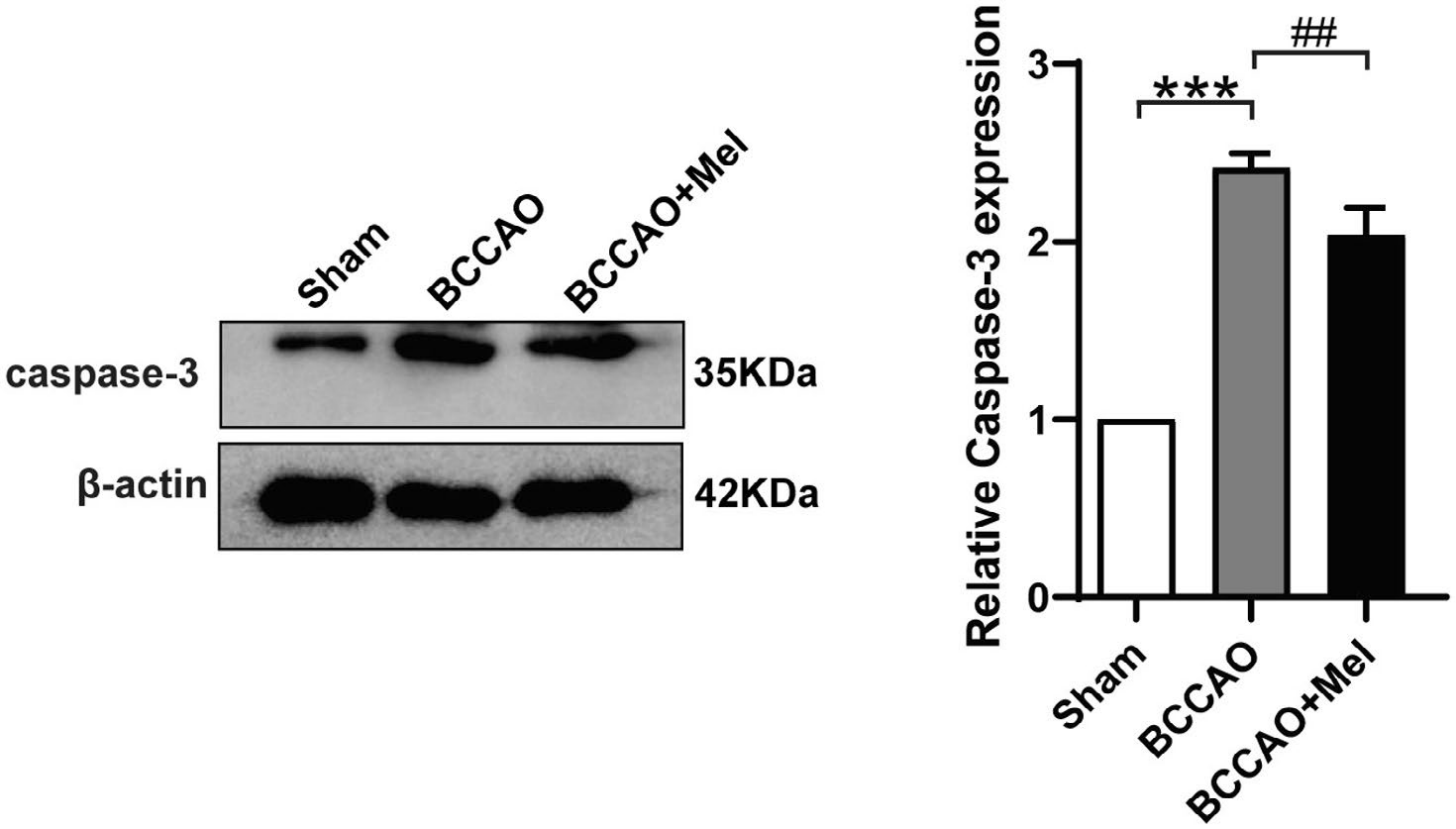
Mel reduced hippocampus caspase‐3 in BCCAO rats, as examined by Western blot (*n* = 3). The Western blot band and quantification of the relative expression of caspase‐3. ***p < 0.001, ^##^ *p* < 0.01.

We quantified the apoptotic protein expression of the key effector, caspase‐3, by Western blot analysis. A marked elevation in caspase‐3 levels was observed in the hippocampus of the BCCAO group relative to Sham‐operated controls (*p* < 0.001; Figure 6). In contrast, caspase‐3 expression in animals that received melatonin treatment (BCCAO + Mel group) was significantly attenuated compared to the BCCAO group (*p* < 0.01; Figure 6). These findings demonstrate that melatonin administration has the potential to reduce the activation of this pro‐apoptotic mediator, supporting its neuroprotective properties in the context of CCH.

## 4 Discussion

The pathophysiology of VaD is recognized as multifactorial, encompassing a spectrum of interrelated cellular and molecular disturbances. Key mechanisms implicated include central cholinergic system dysregulation, synaptic dysfunction, BBB disruption, oxidative stress, neuroinflammation, mitochondrial impairment, aberrant intracellular calcium homeostasis, and the activation of apoptotic pathways [2, 19, 29]. Consistent with this multifactorial pathophysiology, a range of pharmacological agents have demonstrated efficacy in preclinical studies by targeting one or more of these key pathways, leading to improved cognitive performance in animal models of CCH, including the BCCAO model of VaD [16, 21, 22]. To ensure precise surgical intervention and optimize model consistency, BCCAO procedures were performed on rats under a surgical stereomicroscope. Following this, animals in the treatment group received daily intraperitoneal injections of melatonin for 14 consecutive days. In this study, data were collected to evaluate two primary outcome domains: (1) rCBF dynamics, and (2) spatial learning and memory function. In agreement with prior findings from the literature [16], our analyses confirmed the presence of heightened mitophagic activity and increased levels of activated caspase‐3 in the brains of rats subjected to the CCH model of VaD.

The BCCAO model in rats is considered a classic and physiologically relevant paradigm for investigating VaD. This relevance stems from the relatively well‐developed circle of Willis in rodents [26], which facilitates gradual hemodynamic compensation via the posterior circulation following occlusion of the anterior blood supply [30]. Following surgical occlusion, a characteristic biphasic cerebral hemodynamic profile is observed in this model. rCBF typically undergoes an acute, pronounced reduction within 2 to 3 days post‐procedure. This initial drop is succeeded by a sustained phase of CCH, which can persist for a period of approximately 8 to 12 weeks [31]. Following BCCAO in rats, CBF immediately declined to 30%–40% of baseline [30]. In a two‐vessel occlusion (2VO) rat model, frontal cortical CBF recorded by laser‐Doppler flowmetry decreased to approximately 60% of baseline on day 1 post‐surgery and increased to about 65% on day 14 post‐surgery [32]. Although the detection tools and methods varied across studies, the observed changes in CBF following BCCAO were essentially consistent between previous reports and the present study [33, 34]. As previously detailed, high‐resolution laser Doppler imaging was employed to capture cerebral perfusion maps and to quantify the mean blood flow within a defined ROI. This ROI encompassed the hippocampal formation and adjacent cortical areas. The acquired perfusion images provided visual confirmation of successful BCCAO. Subsequent quantitative analysis of these images validated the significant reduction in rCBF resulting from BCCAO, as well as the partial restoration of perfusion following melatonin treatment (Figures 1 and 2). To the best of our knowledge, the specific effects of melatonin on cerebral hemodynamics and the underlying molecular mechanisms in the context of CCH, as modeled by BCCAO, remain insufficiently explored. Based on emerging evidence, the potential mechanisms for such effects are likely to involve the attenuation of neuroinflammatory signaling and the preservation of BBB integrity, both of which are known to be modulated by melatonin [22, 29]. A prior clinical investigation reported that a single intravenous bolus of 10 μg melatonin in human subjects significantly increased peripheral blood flow but did not alter cerebral perfusion. This finding suggests that melatonin does not exert an acute vasoregulatory effect on the cerebral vasculature in humans [35]. However, whether chronic melatonin administration could modulate CBF in patients with VaD remains an open question. Consequently, the potential of melatonin and its receptor agonists to improve CBF in VaD represents a compelling objective for future clinical research.

Pyramidal neurons within the hippocampal CA1 subfield exhibit a selective vulnerability to ischemic injury, a pathophysiological feature that is evolutionarily conserved from rodents to humans [36, 37]. In the present study, CCH induced by BCCAO resulted in functional impairment of the hippocampus and associated neural networks in rats. These deficits were manifested behaviorally as significantly compromised performance across both the acquisition and probe trial phases of the MWM. Notably, melatonin treatment in BCCAO rats effectively ameliorated these cognitive impairments. This was evidenced by a reduction in escape latency during training, alongside an increase in both the number of platform‐site crossings and the percentage of time spent searching in the target quadrant during the probe trial. Together, these behavioral improvements are consistent with previous reports that indicated melatonin administration could enhance spatial learning and memory functions in VaD rats [21, 22].

Mitophagy, a selective form of macroautophagy for the clearance of aging or damaged mitochondria, is vital for maintaining mitochondrial and metabolic homeostasis [38]. There are few studies on mitophagy in VaD, and the existing research results are controversial. Zhao et al. found that activating mitophagy mediated by PINK1/Parkin in the hippocampal CA1 sector could protect VaD rats from ischemic injury [39], whereas Xiao et al. supported that suppressing excessive mitophagy, relieving lysosomal pressure, and reducing free radical generation could play a neuroprotective role on CCH [16]. The discrepancy in mitophagy between the two studies may be attributable to differences in sampling time points, which precisely reflects the dynamic temporal nature of mitophagy. In a more prolonged CCH model, it has been reported that CCH induces a blockade of mitophagic flux, characterized by abnormal accumulation of autophagosomes and impaired degradation, ultimately leading to autophagic dysfunction [40]. Lower lysosomal‐associated membrane protein 1 (LAMP1) expression levels in CCH led to the obstruction of autolysosome formation; further, dysfunctional mitochondria wrapped in autophagosomes failed to degrade [40]. With the accumulation of mitophagy‐mediated autophagosomes, substantial amounts of free radicals were generated from damaged mitochondria, which in turn further triggered mitophagy activation [16]. Compared with the three reports above, the two‐week time point in this study is relatively early. In the early stage of ischemia, mitophagy is strongly activated [6], at which stage lysosomal function remains compensatory to maintain intact autophagy and mitophagy flux. Our study showed that both autophagy and mitophagy were upregulated and synchronized in the hippocampus under two‐week CCH, as evidenced by decreased expression levels of the mitochondrial outer (TOMM20) and inner membrane (COX IV), and increased colocalization of TOMM20 with LC3B.

Melatonin possesses potent antioxidant, anti‐inflammatory, and anti‐apoptotic properties and has demonstrated beneficial effects in VaD, ischemia–reperfusion injury, and neurodegenerative disorders [41]. In the present study, chronic administration of melatonin to BCCAO‐subjected rats inhibited excessive mitophagic activity. This effect was characterized molecularly by a reduced LC3B‐II/LC3B‐I ratio (an autophagic marker) and increased expression of the mitochondrial proteins TOMM20 and COX IV, findings that are consistent with previous reports. A recent preclinical investigation demonstrated that melatonin administration attenuates excessive mitophagic activity in a murine model of post‐stroke cognitive impairment, and this protective effect is mechanistically linked to the modulation of the PINK1/Parkin signaling pathway [42]. Although the present study did not investigate the underlying mechanisms, previous findings suggest that melatonin may modulate mitophagy indirectly, for instance by mitigating excessive oxidative stress and thereby protecting mitochondria from further damage. Melatonin was found to suppress oxidative stress in a 2VO rat model, as evidenced by decreased levels of malondialdehyde (MDA) and NADPH oxidase 4 (NOX4) [21]. Moreover, melatonin may promote autophagosome‐lysosome fusion, thereby influencing mitophagy. In HT22 cells exposed to oxygen–glucose deprivation followed by reoxygenation (OGD/R), melatonin was found to increase Rab7 expression, which facilitates autophagolysosome formation [43]. Thus, melatonin is thought to promote efficient removal of damaged mitochondria and to prevent the buildup of dysfunctional mitochondria that could otherwise perpetuate the vicious cycle of excessive mitophagy in VaD. Concurrently, CCH impairs mitochondrial permeability, leading to substantial release of cytochrome c and reactive oxygen species (ROS), which in turn drives mitophagy and activates caspase‐9 and caspase‐3 [2, 44]. Melatonin has been shown to prevent cytochrome c release and to preserve mitochondrial integrity [45]. Based on the various mechanisms of melatonin in the treatment of VaD models, many studies have reported its anti‐apoptotic and neuroprotective effects [21, 22, 42]. Our study also showed that melatonin treatment downregulated the expression of the apoptotic executor caspase‐3.

The study has some limitations. First, the investigation was conducted exclusively at a single time point (2 weeks post‐surgery). Given that CCH can persist for 8–12 weeks in the BCCAO model, the temporal dynamics of mitophagy, including whether it is insufficient, appropriate, or excessive at different stages, remain to be fully characterized. The optimal timing for therapeutic intervention also requires further studies. Second, although alterations in mitochondrial membrane proteins (TOMM20 and COX IV) and autophagosomal markers (LC3B) were observed, the upstream signaling pathways that regulate mitophagy were not investigated. Third, loss‐ or gain‐of‐function experiments were not performed to determine whether the cognitive improvements induced by melatonin are directly mediated by mitophagy suppression. Future studies employing pharmacological or genetic manipulations are warranted to validate this potential causal link.

## 5 Conclusion

This study provides evidence for heightened mitophagic activity in the hippocampus of a rodent model of VaD. The administration of melatonin was found to ameliorate cognitive deficits in these animals. This therapeutic effect correlates with a multifaceted mechanism involving the facilitation of CBF recovery, the attenuation of excessive mitophagy, and the downregulation of pro‐apoptotic caspase‐3 expression. Collectively, these findings contribute novel mechanistic insights that may inform the development of future therapeutic strategies for VaD.

## Author Contributions

Conceptualization: Yang Li and Sui‐yi Xu; data curation: Xing‐yi Guo, Zhi‐gang Wang, and Ting‐yan Chen; formal analysis: Si‐jia Hou and Xing‐yi Guo; investigation: Yang Li; methodology: Si‐jia Hou, Xing‐yi Guo, Zhi‐gang Wang, Ting‐yan Chen, and SX; project administration: Yang Li; resources: Sui‐yi Xu; supervision: Yang Li and Sui‐yi Xu; writing – original draft: Si‐jia Hou and SX; writing – review and editing: Si‐jia Hou and Sui‐yi Xu. All authors approved the final version of the manuscript.

## Funding

This work was supported by the Shanxi Applied Basic Research Program (202303021221222).

## Ethics Statement

All experimental procedures were approved by the Ethics Committee of the First Hospital of Shanxi Medical University (DWLL‐2024‐004).

## Conflicts of Interest

The authors declare no conflicts of interest.

## Supporting information


**Figure S1:** Target quadrant crossings on day 6 of the MWM test (*n* = 5). **p < 0.01, ^#^ *p* < 0.05.

## Data Availability

The datasets generated during and/or analyzed during the current study are available from the corresponding author on reasonable request.
